# Effect of e-health interventions on HIV prevention: a protocol of systematic review and meta-analysis

**DOI:** 10.1186/s13643-023-02274-6

**Published:** 2023-06-30

**Authors:** Lei Wang, Xiang-yu Yan, Lin Mei, Zhong-wei Jia, Rui-gang Hao, Ji-hong Xu, Bo Zhang

**Affiliations:** 1 Taiyuan Center for Disease Control and Prevention, Taiyuan, Shanxi China; 2 School of Public Health, Peking University, Beijing, China; 3 Institute of Disaster and Emergency Medicine, Tianjin University, Tianjin, China; 4 Center for Intelligent Public Health, Institute for Artificial Intelligence, Peking University, Beijing, China; 5 Center for Drug Abuse Control and Prevention, National Institute of Health Data Science, Peking University, Beijing, China; 6 School of Life Sciences, Hainan University, Haikou, 570228 China

**Keywords:** E-health, Social media, Intervention, Human immunodeficiency virus, Acquired immunodeficiency syndrome, Prevention

## Abstract

**Background:**

Global epidemiological data indicates that despite implementation of multiple interventions and significant financial investment, the HIV/AIDS epidemic remained inadequately controlled as of 2020. E-health presents a novel approach in delivering health information and health care and has gained popularity in HIV prevention worldwide. However, evidence on the effectiveness of e-health interventions on HIV prevention among diverse populations remains inadequate. Our study aims to systematically evaluate the effectiveness of varying e-health interventions on HIV prevention, with the objective of providing data support and guidance for the development of future e-health HIV intervention strategies.

**Methods:**

A systematic search of electronic English databases, including MEDLINE through PubMed, Embase, Scopus, and Web of Science, along with three Chinese databases, including National Knowledge Infrastructure (CNKI), Chinese Wanfang Digital Periodicals (WANFANG), and Chinese Science and Technology Periodicals (VIP) database, will be conducted for the period of 1 January 1980 to 31 December 2022. Additionally, gray literature and unpublished trials in trial registers will be searched. Studies aimed at HIV prevention through e-health interventions, with full-text publications available in either English or Chinese, will be included. Study types will be limited to RCT, cluster RCT, and quasi-experiment study. The risk of bias in individual studies will be assessed following the guideline highlighted by the Cochrane Handbook for Systematic Reviews of Interventions. The outcomes will cover cognitive, behavioral, psychological, management, and biological measures of individuals involved in e-health interventions. The quality of evidence will be assessed by the Grading of Recommendations, Assessment, Development, and Evaluation (GRADE) approach. Ultimately, a systematic review with meta-analysis will be conducted to compare the effectiveness of e-health interventions among diverse populations.

**Discussion:**

This systematic review seeks to establish novel insights into the effectiveness of e-health interventions in diverse populations worldwide. It will inform the design and use of e-health interventions to optimize HIV-related strategies.

**Systematic review registration:**

PROSPERO CRD42022295909.

**Supplementary Information:**

The online version contains supplementary material available at 10.1186/s13643-023-02274-6.

## Background

The HIV epidemic has presented significant global challenges, prompting a major focus on HIV prevention as a public health priority. In this context, the United Nations has established ambitious goals, including the end of the AIDS epidemic by 2030 [1], as well as the objective of reducing new HIV infections to fewer than 500,000 annually [2]. Despite the efforts of the international health community, these goals remain elusive. To illustrate, the United Nations Program on HIV and AIDS (UNAIDS) has reported that, as of 2021, approximately 38.4 million people worldwide are living with HIV [3]. Moreover, there were about 1.5 million new HIV infections in 2021, which was a number far exceeding the targeted goal and has threatened not only global public health but also society and the economy [3].

Despite the implementation of numerous intervention strategies such as immediate start of antiretroviral therapy (ART), promotion of condom uses, opioid drug substitution, needle and syringe exchange program, and the provision of voluntary consultation and testing (VCT) services, targeted interventions for key populations, school-based awareness campaigns, promotion of HIV self-testing [4, 5], the decline in new HIV infections remains insufficient for achieving established targets. Moreover, HIV transmission patterns have recently shifted from high-risk populations to the general population worldwide [6–8]. Given the increasing public health burden of HIV and the pressing need to end the epidemic by 2030, innovative interventions must be integrated into traditional approaches due to changes in people’s lifestyle and behaviors of seeking sexual partners.

The proliferation of the Internet and the increasing emergence of dating apps or websites have significantly enhanced the ease and privacy of meeting and approaching causal sexual partners, potential romantic partners, one-night stand partners, and Internet partners [9–12]. Internet friendship platforms, such as some geosocial networking applications (GSN apps) especially dating apps (e.g., Tinder, Grindr), have made communication and interaction with other users in close proximity extremely convenient [13]. While public venues such as bars and parks were traditionally popular haunts for seeking causal sexual partners in the past, the use of GSN apps or dating apps has increasingly replaced these means of courtship in recent years [14]. Gravningen K. et al.’s study showed that 30% of Norwegian adolescents reported Internet partner seeking by using dating apps (e.g., Tinder, Bumble, Hinge) and social media networks (e.g., Facebook, Twitter, Instagram) [12]. Studies have shown that a significant number of men who have sex with men (MSM) in the USA, ranging from 36.0 to 63.6%, and mainland China (40.6%) used GSN apps to seek male partners [15–18]. Furthermore, HIV incidence among GSN apps’ users was found to be 4 times higher than that of nonusers (8.5 vs 2.0 per 100 person-years) [19]. Therefore, there is an urgent need for targeted HIV prevention measures through Internet.

E-health is defined as a collection of electronic technologies that utilize the Internet to provide healthcare services, thereby improving the quality of life and facilitating healthcare delivery [20, 21]. These technologies encompass a variety of practices such as electronic medical records (eMRs), electronic health records (eHRs), mobile health (m-health) applications for health practice, remote service provision through telecommunications (telehealth and telemedicine), electronic health information systems (eHIS), medication systems, and social media platforms [22]. The World Health Organization (WHO) recognizes that e-health has the potential to strengthen preventive medical care, enhance healthcare quality, reduce costs for healthcare institutions and users, and increase access to healthcare services for poor, underserved, vulnerable populations and people in marginalized areas [20]. Furthermore, e-health has the potential to transform healthcare service access and quality and help contain costs [23]. The WHO emphasizes that e-health plays a key role in achieving universal health coverage [24].

Currently, there is increasing academic interest in the effectiveness of e-health interventions for HIV prevention worldwide. For example, Marhefka, Turner, and Lockhart [25] utilized an e-health video conference program for women living with HIV (WLH), investigating the feasibility of group-based e-health interventions. Michael Argenyi et al. [26] utilized social media in HIV screening outreach for deaf and hard-of-hearing adults, demonstrating its effectiveness in mitigating technological or linguistic barriers. David Loutfi et al. [27] analyzed a pilot social media intervention for HIV prevention among marginalized young women in Botswana, revealing that while social media could enhance reach to hard-to-reach populations, its acceptance was lower than that of face-to-face interventions. Furthermore, Brooks, Nieto, Swendeman, Myers, Lepe, Cabral, Kao, Donohoe, and Comulada [28] reported that social media and mobile technology (SMMT) interventions were well accepted in managing HIV care among youth and young adults aged 13–34 living with HIV. Overall, previous studies on e-health HIV interventions were varied in terms of population and intervention tools, and the findings were often contradictory across studies. Therefore, there is a critical need to synthesize and integrate the existing evidence to establish more robust conclusions.

Nevertheless, previous systematic reviews on this topic have provided incomplete evidence. On one hand, most reviews have only focused on promoting treatment among persons living with HIV, disregarding the effectiveness of e-health in preventing HIV infection [29–31]. On the other hand, previous relevant systematic reviews have predominantly concentrated on key populations, such as MSM [32, 33], African countries with a high burden of HIV epidemic [34–36], and HIV-related outcomes, while disregarding other outcomes such as psychological health [37]. To end the AIDS pandemic by 2030, all population strategies must be considered. However, globally, there is a paucity of systematic summaries and classification of previous evidence regarding e-health intervention effectiveness for all populations. Hence, the objective of this study is to systematically summarize and quantitatively integrate different types of e-health interventions’ effectiveness across various populations and regions, enabling the selection for more effective prevention measures to end AIDS by 2030.

## Methods

### Protocol and registration

A systematic review with meta-analysis will be implemented in this study. This protocol of meta-analysis will be performed on the basis of the Preferred Reporting Items for Systematic review and Meta-Analysis Protocol (PRISMA-P) statement (Additional file 1) [38], and the reporting of the following systematic review with meta-analysis will use the PRISMA extension statement as a guide. This study has been registered at PROSPERO with registration number CRD42022295909.

### Eligibility criteria and type of study

The optimal study design for inclusion in this systematic review is randomized controlled trials (RCTs). However, considering the inherent characteristics of public health interventions, individualized randomization may be impractical in some circumstances. Therefore, cluster RCTs and quasi-experimental studies with self-control will also be considered for inclusion. Cross-sectional studies utilizing only online surveys will not be incorporated in this analysis. Studies that meet the following inclusion criteria will be included: (1) focused on HIV prevention, (2) the study conducting an e-health intervention originally, (3) written in English and retrieved from electronic English databases or in Chinese and retrieved from electronic Chinese databases with full-text access, and (4) published within the timeframe of January 1, 1980, to December 31, 2022, as the first documented case of HIV was reported in Los Angeles in 1981 [39].

### Participants

In this study, participants who received an e-health intervention will be included. The subgroup analysis will primarily concentrate on evaluating the effectiveness of e-health interventions among university students, women, adolescents, and HIV key populations (i.e., men who have sex with men, sex workers, people who inject drugs, transgender individuals, and individuals confined in correctional facilities).

### Type of interventions

Original researches conducting e-health interventions on HIV prevention will be included. E-health interventions are defined as those implemented through the Internet, such as m-health and telehealth, which utilize electronic technologies to provide healthcare resources, services, and information. Studies that solely conducted online surveys without any intervention will be excluded from the analysis.

### Outcomes of interest

We will consider the systematic reviews with the following outcomes; the details of these outcomes are shown in Table 1.

**Table 1:** Outcomes of interest

Classification	Outcomes	Variable type
Cognitive outcomes	1) HIV-related knowledge	Continuous
	Self-efficacy	Continuous
	3) Discriminate against HIV	Continuous
	Acceptance to HIV-infected person	Continuous
	Attitudes towards condom use	Continuous
	Attitudes towards susceptibility to HIV	Continuous
	PrEP (pre-exposure prophylaxis)/PEP (postexposure prophylaxis) awareness	Continuous
	Others found in data extraction	
Behavior outcomes	1) Condom use	Binary
	2) Frequency in unprotected sexual intercourse	Continuous
	3) Number of sexual partners	Continuous
	4) Initiation of ART	Binary
	Attendance to HIV testing	Binary
	HIV counselling	Binary
	Uptake of medical male circumcision	Binary
	Uptake PrEP	Binary
	Uptake PEP	Binary
	Others found in data extraction	
Psychological health outcomes	1) Severity of anxiety and depression	Continuous
	2) Social support	Continuous
	3) Others found in data extraction	
Management outcomes	1) Acceptability to intervention	Binary
	2) Retention in HIV care	Binary
	3) Adherence to ART	Binary
	Attendance at pharmacy visit	Binary
	5) Others found in data extraction	
Biological outcomes	HIV infection rate	Binary
	2) Opportunistic infection rate	Binary
	Mean CD4 cell count	Continuous
	Proportion of participants virally suppressed	Binary
	Others found in data extraction	

### Data sources and search strategy

#### Electronic searches

We plan to conduct a comprehensive and systematic search of various electronic English databases, including MEDLINE, PubMed, Embase, Scopus, and Web of Science, as well as three Chinese databases, which are National Knowledge Infrastructure (CNKI), Chinese Wanfang Digital Periodicals (WANFANG), and Chinese Science and Technology Periodicals (VIP) database. The detail of these search strategies is shown in Additional file 2. This search strategy has been collaboratively developed by a medical librarian and all authors and is based on key terms from previous literature. In addition to this, we will perform a thorough examination of the reference lists of identified relevant RCTs and reviews, contact experts in the field of HIV e-health interventions to identify any additional trials or results, and scrutinize ClinicalTrials.gov, Chinese Clinical Trial Registry (ChiCTR), and International Clinical Trials Registry Platform (ICTRP) to identify planned, ongoing, or unpublished trials. In order to retrieve any gray literature, we will also search Google Scholar and Baidu Scholar.

### Study selection

**Fig. 1:**
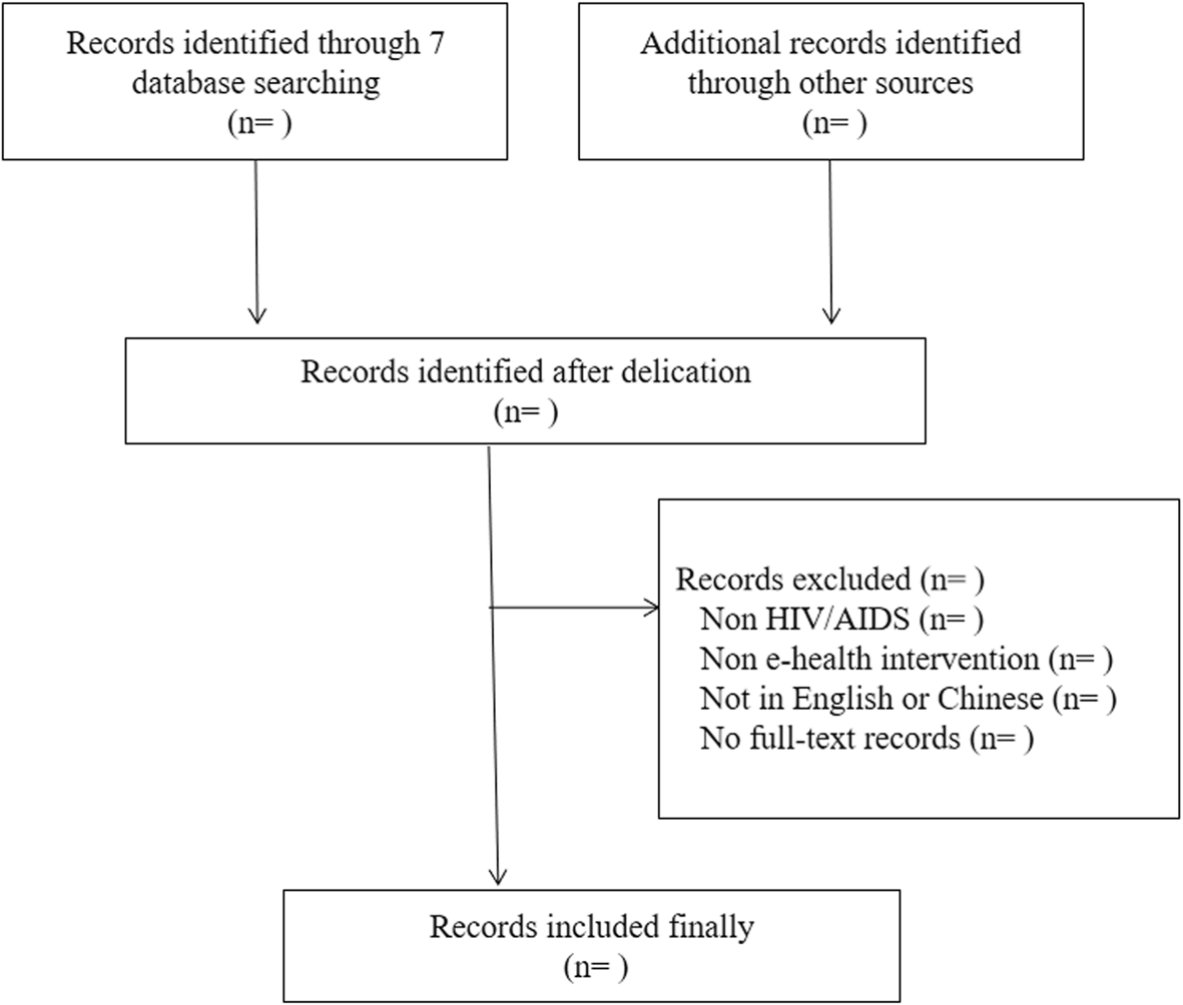
PRISMA 2009 flow chart

Two reviewers (L. W. and X. Y.) will independently assess the titles and abstracts that meet the initial retrieval criteria. Studies that do not satisfy the eligibility criteria will be excluded. Subsequently, the remaining publications will undergo a full-text screening by both investigators (L. W. and X. Y.) according to the same inclusion criteria. In the event of any disagreements, discussion will be employed to seek consensus. Any discrepancy that cannot be resolved will be referred to a third reviewer to determine the final decision. Furthermore, excluded publications and their respective reasons for elimination will be confirmed by a third reviewer. A PRISMA 2009 flow chart (Fig. 1) will be used to show the process of study selection. The extracted references will be managed using EndNote software (version X9 Windows). A pilot test using articles published in 2022 will be conducted, and necessary adjustments will be made based on the results.

#### Data extraction

The studies selected through the study selection process will undergo data extraction, wherein information from the studies will be extracted after a thorough reading of the full text, and presented in Table 2. The data extraction form will be created using Microsoft Excel 2016.

**Table 2:** Data extraction form

Primary item	Secondary item
Basic information of study	1) Title
	2) Author
	3) Publish year
	4) Country
	5) Study type
	6) Others found in data extraction
E-health interventions	1) Intervention name and description
	2) Social media platform
	3) Control group
	4) Target population
	5) Number of participants (total number, number of each arm and subgroup, etc.)
	6) Time duration of intervention
	7) Others found in data extraction
Effects of intervention^a^	1) Cognitive outcomes
	2) Behavior
	3) Psychological health
	4) Management outcomes
	5) Biological outcomes

^a^Means and SDs or SEs for continuous outcomes and numbers for the respective outcomes/events for dichotomous ones

Data extraction will be conducted by two independent reviewers (L. W. and X. Y.) utilizing the designed data extraction form. Following this process, the records extracted by the reviewers will be cross-checked, and any disputed points will be resolved through a third reviewer who will make the final decision. Upon completion of the extraction process, the data records will be sorted by region and year. This will result in a dataset of included studies for data analysis. A pilot test will be conducted on selected studies published in 2022 with adjustments made as necessary thereafter.

### Risk-of-bias assessment

The Cochrane Collaboration risk-of-bias (ROB) tool or Cochrane risk-of-bias tool for cluster-randomized control trials (RoB2.0) will be used by two independent authors (L. W. and X. Y.) to assess the risk of bias in the included studies [40–42]. In the event of any disagreements, they will be resolved through discussion, and any unresolved disagreement will be referred to the third reviewer for a final decision. Each criteria of RoB or RoB2.0 will be classified into low risk (meet the standard), unclear risk (specific details or descriptions were not reported), or high risk (not fulfilling the criteria). The quality assessment results will be presented using the Cochrane risk-of-bias assessment chart [43].

### Statistical analysis

The extracted data will be reported using descriptive statistics. Meta-analysis will be employed to generate pooled estimates. Relevant indicators will utilize the standardized mean difference (SMD) [44] for continuous variables (e.g., knowledge score, scale score) and relative risk (RR) or odds ratio (OR) for categorical variables (e.g., HIV testing, condom use). All statistical analyses will be conducted using meta-analysis package in R software version 4.2.2 (R Core Team). Inconsistency (*I*^2^) based on the chi-squared test will be utilized to assess overall heterogeneity, classified into three levels: *I*^2^ ≤ 50%, low heterogeneity; *I*^2^ > 50% to *I*^2^ < 75%, moderate heterogeneity; and *I*^2^ ≥ 75%, high heterogeneity. A random-effects model is preferable, particularly when *I*^2^ > 50% [45, 46]. Outcomes will be considered statistically significant when *p* < 0.05. Furthermore, for studies investigating various types of e-health interventions for the same outcome, network meta-analysis using the Bayesian approach will be utilized to compare the effectiveness among different types of e-health interventions if possible. For outcomes that cannot be quantitatively synthesized due to too few studies available or high heterogeneity in intervention methods or study populations, a narrative synthesis will be conducted.

If feasible, subgroup analysis will be conducted to evaluate intervention effects in various subpopulations, including (1) target populations, such as key populations (e.g., MSM, people who inject drugs, people in prisons and other closed settings, sex workers, and transgender people) and other focused population subgroups (e.g., adolescents, women, university students); (2) sex (male, female); (3) age groups; (4) types of e-health interventions; (5) types of countries, encompassing western or eastern countries and developing or developed countries; (6) prevalence states of countries, including those with the highest, moderate, and lowest rates of HIV prevalence; and (7) interactive and noninteractive e-health interventions.

Sensitivity analysis will be conducted to assess the stability and reliability of the study by excluding studies with special characteristics, such as those of lower quality (e.g., before-after studies conducted in the same population). Furthermore, we will perform additional sensitivity analyses by iteratively excluding each study to gauge its impact on overall estimates and to ascertain the stability of results.

Publication bias will be assessed by using a funnel plot (suggested in cases where no less than 10 studies are included in the meta-analysis) and Egger’s test [47]. To test asymmetry in the funnel plot, a rank correlation test or regression analysis will be employed. In circumstances where data is accessible for the analysis from no less than 10 trials, meta-regression techniques will also be employed to investigate the association of trial characteristics with effect sizes.

If cluster-randomized trials are incorporated, we will perform sample size adjustment utilizing estimates to evaluate covariance with cluster-level adjustment for participants with measurements at both baseline and final follow-up. Furthermore, interaction terms will be employed to examine the consistency of effects among subgroups [48, 49].

### Certainty of evidence

The Grading of Recommendations Assessment, Development, and Evaluation (GRADE) will be utilized to assess the level of certainty of evidence [50]. A rating of high, moderate, low, or very low quality will be assigned to each piece of evidence based on its performance across five domains, comprising of risk of bias, inconsistency, indirectness, imprecision, and other relevant considerations [51, 52]. The GRADE-based determination of evidence certainty will be carried out by two independent authors (L. W. and X. Y.), with any disagreements resolved through discussion. For cases where an agreement is not reached, the matter will be referred to a third reviewer for final adjudication.

## Discussion

Based on the protocol of this study, a pioneering systematic review with meta-analysis will be conducted to summarize and assess the impact of e-health interventions on HIV prevention across various outcome indicators and populations worldwide. Although several factors, including imperfect e-health implementation and demographic factors such as gender, residence, income, education, and culture, can influence the adoption of e-health, it still holds significant potential in promoting people’s health status [53]. Our findings will highlight the diverse contributions made by e-health interventions and aid in the development and decision-making of public health strategies for HIV prevention. However, e-health interventions may not show advantages over traditional methods in some aspects, because it changes the communication mode between the intervention implementers and recipients. Hence, it is crucial to capitalize on the advantages while avoiding potential disadvantages. Ultimately, the intended outcome is a range of policy options that employ e-health interventions, promoting an accelerated end to the AIDS pandemic. One limitation of the current study is the inclusion of studies written exclusively in English and Chinese, hence leading to the possibility of missing relevant studies written in other languages. To enhance the comprehensiveness of our study, we will incorporate evidence generated from different types of studies (e.g., RCT, cluster RCT, and quasi-experiment study). Furthermore, we recommend users to adapt or translate the framework within their respective contexts, considering other relevant characteristics such as subgroups, cultural factors, and potential barriers. In summary, this systematic review will bring a novel concept and direction that exploits e-health to achieve the aspirational goal of ending AIDS by 2030.

## Supplementary Information

**Additional file 1. **PRISMA-P 2015 Checklist.

**Additional file 2. **Search strategy.

## Data Availability

Not applicable.

## Abbreviations

UNAIDS: The United Nations Program on HIV and AIDS
RCTs: Randomized controlled trials
ART: Antiretroviral therapy
VCT: Voluntary consultation test
GSN apps: Geosocial networking applications
MSM: Men who have sex with men
eMRs: Electronic medical records
eHRs: Electronic health records
m-health: Applications for health practice for mobile health
eHIS: Electronic health information systems
WHO: World Health Organization
SMMT: Social media and mobile technology
PRISMA-P: Preferred Reporting Items for Systematic review and Meta-Analysis Protocol
PrEP: Pre-exposure prophylaxis
PEP: Postexposure prophylaxis
ROB: The Cochrane Collaboration risk-of-bias (ROB) tool
RoB2.0: Cochrane risk-of-bias tool for cluster-randomized control trials
SMD: Standardized mean difference
SD: Standard deviation
SE: Standard error
RR: Relative risk
OR: Odds ratio
GRADE: Grading of Recommendations Assessment, Development, and Evaluation
ChiCTR: Chinese Clinical Trial Registry
ICTRP: International Clinical Trials Registry Platform
