# Supplementary material for: Effect of e-health interventions on HIV prevention: a protocol of systematic review and meta-analysis
Source: Syst Rev. 2023 Jun 30;12:106. doi: 10.1186/s13643-023-02274-6 (PMC10311723; doi:10.1186/s13643-023-02274-6)
Supplement: Supplementary file 2 — Additional file 2. Search strategy. [file 13643_2023_2274_MOESM2_ESM.docx]

**Search strategy**

1. English database

1.1 Pubmed

(((("hiv"[MeSH Terms] OR "human immunodeficiency virus"[Title/Abstract] OR "human immunodeficiency viruses"[Title/Abstract] OR "AIDS"[Title/Abstract] OR "acquired immuno-deficiency syndrome"[Title/Abstract] OR "acquired immunodeficiency syndrome"[MeSH Terms] OR "acquired immune deficiency syndrome"[Title/Abstract]) AND ("social media"[Title/Abstract] OR "social medium"[Title/Abstract] OR "communications media"[Title/Abstract] OR "computer communication network"[Title/Abstract] OR "internet"[Title/Abstract] OR "digital communication technology"[Title/Abstract] OR "digital technology"[Title/Abstract] OR "online"[Title/Abstract] OR "blogging"[Title/Abstract] OR "webcast"[Title/Abstract] OR "web"[Title/Abstract] OR "health information"[Title/Abstract] OR "medical information"[Title/Abstract] OR "ehealth"[Title/Abstract] OR "e-health"[Title/Abstract] OR "electronic health"[Title/Abstract] OR "mHealth"[Title/Abstract] OR "mobile health"[Title/Abstract] OR "telemedicine"[Title/Abstract] OR "telecommunication"[Title/Abstract] OR "e-mail"[Title/Abstract] OR "email"[Title/Abstract] OR "application"[Title/Abstract] OR "app"[Title/Abstract] OR "social web"[Title/Abstract] OR "social network"[Title/Abstract] OR "social networking"[Title/Abstract] OR "geosocial networking"[Title/Abstract] OR "geosocial network"[Title/Abstract] OR "gsn application"[Title/Abstract] OR "gsn app"[Title/Abstract] OR "smartphone"[Title/Abstract] OR "mobile app"[Title/Abstract] OR "mobile application"[Title/Abstract] OR "software application"[Title/Abstract] OR "software app"[Title/Abstract] OR "social software"[Title/Abstract] OR "social gaming"[Title/Abstract] OR "blog"[Title/Abstract] OR "podcast"[Title/Abstract] OR "Facebook"[Title/Abstract] OR "web log"[Title/Abstract] OR "YouTube"[Title/Abstract] OR "twitter"[Title/Abstract] OR "tweet"[Title/Abstract] OR "gay apps"[Title/Abstract] OR "microblogging"[Title/Abstract] OR "vodcast"[Title/Abstract] OR "google plus"[Title/Abstract] OR "Google"[Title/Abstract] OR "Instagram"[Title/Abstract] OR "LinkedIn"[Title/Abstract] OR "Pinterest"[Title/Abstract] OR "Reddit"[Title/Abstract] OR "second life"[Title/Abstract] OR "Tumblr"[Title/Abstract] OR "Weibo"[Title/Abstract] OR "WeChat"[Title/Abstract] OR "Grindr"[Title/Abstract] OR "Jack'd"[Title/Abstract] OR "Scruff"[Title/Abstract] OR "Growlr"[Title/Abstract] OR "OkCupid"[Title/Abstract] OR "QQ"[Title/Abstract] OR "Qzone"[Title/Abstract] OR "Skype"[Title/Abstract] OR "Snapchat"[Title/Abstract] OR ("VKontakte"[Title/Abstract]) OR "Whatsapp"[Title/Abstract] OR "baidu tieba"[Title/Abstract] OR "Viber"[Title/Abstract] OR "Zank"[Title/Abstract] OR "Hornet"[Title/Abstract] OR "Messenger"[Title/Abstract] OR "Blued"[Title/Abstract] OR "Tinder"[Title/Abstract] OR "TanTan"[Title/Abstract] OR "MOMO"[Title/Abstract] OR "Soul"[Title/Abstract] OR "Gpark"[Title/Abstract] OR "Aloha"[Title/Abstract])) AND ("promot*"[Title/Abstract] OR "prevent*"[Title/Abstract] OR "interven*"[Title/Abstract] OR "implement*"[Title/Abstract] OR "evaluat*"[Title/Abstract] OR "effect*"[Title/Abstract])) AND (English[Language])) AND (("1980/01/01"[Date - Completion] : "2022/12/31"[Date - Completion]))

1.2 EmBase

#1 'hiv'/exp OR 'aids'/exp OR 'human immunodeficiency virus':ab,ti OR 'human immunodeficiency viruses':ab,ti OR 'acquired immunodeficiency syndrome':ab,ti OR 'acquired immune deficiency syndrome':ab,ti OR 'acquired immuno-deficiency syndrome':ab,ti

#2 'social medium':ab,ti OR 'communications media':ab,ti OR 'computer communication network':ab,ti OR internet:ab,ti OR 'digital communication technology':ab,ti OR 'digital technology':ab,ti OR online:ab,ti OR blogging:ab,ti OR webcast:ab,ti OR web:ab,ti OR 'medical information':ab,ti OR 'e health':ab,ti OR ehealth:ab,ti OR 'electronic medical':ab,ti OR mhealth:ab,ti OR 'mobile health':ab,ti OR telemedicine:ab,ti OR telecommunication:ab,ti OR 'e mail':ab,ti OR email:ab,ti OR application:ab,ti OR app:ab,ti OR 'social web':ab,ti OR 'social networking':ab,ti OR 'geosocial networking':ab,ti OR 'geosocial network':ab,ti OR 'gsn application':ab,ti OR 'gsn app':ab,ti OR smartphone:ab,ti OR 'mobile app':ab,ti OR 'mobile application':ab,ti OR 'software application':ab,ti OR 'software app':ab,ti OR 'social software':ab,ti OR 'social gaming':ab,ti OR blog:ab,ti OR podcast:ab,ti OR facebook:ab,ti OR 'web log':ab,ti OR 'social media':ab,ti OR twitter:ab,ti OR tweet:ab,ti OR 'gay apps':ab,ti OR microblogging:ab,ti OR vodcast:ab,ti OR 'google plus':ab,ti OR google+:ab,ti OR instagram:ab,ti OR linkedin:ab,ti OR pinterest:ab,ti OR reddit:ab,ti OR 'second life':ab,ti OR tumblr:ab,ti OR weibo:ab,ti OR 'social media analysis':ab,ti OR grindr:ab,ti OR jackd:ab,ti OR scruff:ab,ti OR growlr:ab,ti OR okcupid:ab,ti OR qq:ab,ti OR qzone:ab,ti OR skype:ab,ti OR snapchat:ab,ti OR 'v kontakte':ab,ti OR whatsapp:ab,ti OR 'baidu tieba':ab,ti OR viber:ab,ti OR zank:ab,ti OR hornet:ab,ti OR messenger:ab,ti OR blued:ab,ti OR tinder:ab,ti OR tantan:ab,ti OR momo:ab,ti OR soul:ab,ti OR gpark:ab,ti OR aloha:ab,ti

#3 promot*:ab,ti OR prevent*:ab,ti OR interven*:ab,ti OR implement*:ab,ti OR evaluat*:ab,ti OR effect*:ab,ti

#4 english:la

#5 [1-1-1980]/sd NOT [31-12-2022]/sd

#6 #1 AND #2 AND #3 AND #4 AND #5

1.3 Scopus

( TITLE ( "hiv" ) OR ABS ( "hiv" ) OR TITLE ( "human immunodeficiency virus" ) OR ABS ( "human immunodeficiency virus" ) OR TITLE ( "human immunodeficiency viruses" ) OR ABS ( "human immunodeficiency viruses" ) OR TITLE ( "aids" ) OR ABS ( "aids" ) OR TITLE ( "acquired immunodeficiency syndrome" ) OR ABS ( "acquired immunodeficiency syndrome" ) OR TITLE ( "acquired immune deficiency syndrome" ) OR ABS ( "acquired immune deficiency syndrome" ) OR TITLE ( "acquired immuno-deficiency syndrome" ) OR ABS ( "acquired immuno-deficiency syndrome" ) ) AND ( TITLE ( "social media" ) OR ABS ( "social media" ) OR TITLE ( "social medium" ) OR ABS ( "social medium" ) OR TITLE ( "communications media" ) OR ABS ( "communications media" ) OR TITLE ( "computer communication network" ) OR ABS ( "computer communication network" ) OR TITLE ( "internet" ) OR ABS ( "internet" ) OR TITLE ( "digital communication technology" ) OR ABS ( "digital communication technology" ) OR TITLE ( "digital technology" ) OR ABS ( "digital technology" ) OR TITLE ( "online" ) OR ABS ( "online" ) OR TITLE ( "blogging" ) OR ABS ( "blogging" ) OR TITLE ( "webcast" ) OR ABS ( "webcast" ) OR TITLE ( "web" ) OR ABS ( "web" ) OR TITLE ( "health information" ) OR ABS ( "health information" ) OR TITLE ( "medical information" ) OR ABS ( "medical information" ) OR TITLE ( "ehealth" ) OR ABS ( "ehealth" ) OR TITLE ( "e-health" ) OR ABS ( "e-health" ) OR TITLE ( "electronic health" ) OR ABS ( "electronic health" ) OR TITLE ( "mhealth" ) OR ABS ( "mhealth" ) OR TITLE ( "mobile health" ) OR ABS ( "mobile health" ) OR TITLE ( "telemedicine" ) OR ABS ( "telemedicine" ) OR TITLE ( "telecommunication" ) OR ABS ( "telecommunication" ) OR TITLE ( "e-mail" ) OR ABS ( "e-mail" ) OR TITLE ( "email" ) OR ABS ( "email" ) OR TITLE ( "application" ) OR ABS ( "application" ) OR TITLE ( "app" ) OR ABS ( "app" ) OR TITLE ( "social web" ) OR ABS ( "social web" ) OR TITLE ( "social network" ) OR ABS ( "social network" ) OR TITLE ( "social networking" ) OR ABS ( "social networking" ) OR TITLE ( "geosocial networking" ) OR ABS ( "geosocial networking" ) OR TITLE ( "geosocial network" ) OR ABS ( "geosocial network" ) OR TITLE ( "gsn application" ) OR ABS ( "gsn application" ) OR TITLE ( "gsn app" ) OR ABS ( "gsn app" ) OR TITLE ( "smartphone" ) OR ABS ( "smartphone" ) OR TITLE ( "mobile app" ) OR ABS ( "mobile app" ) OR TITLE ( "mobile application" ) OR ABS ( "mobile application" ) OR TITLE ( "software application" ) OR ABS ( "software application" ) OR TITLE ( "software app" ) OR ABS ( "software app" ) OR TITLE ( "social software" ) OR ABS ( "social software" ) OR TITLE ( "social gaming" ) OR ABS ( "social gaming" ) OR TITLE ( "blog" ) OR ABS ( "blog" ) OR TITLE ( "podcast" ) OR ABS ( "podcast" ) OR TITLE ( "facebook" ) OR ABS ( "facebook" ) OR TITLE ( "web log" ) OR ABS ( "web log" ) OR TITLE ( "youtube" ) OR ABS ( "youtube" ) OR TITLE ( "twitter" ) OR ABS ( "twitter" ) OR TITLE ( "tweet" ) OR ABS ( "tweet" ) OR TITLE ( "gay apps" ) OR ABS ( "gay apps" ) OR TITLE ( "microblogging" ) OR ABS ( "microblogging" ) OR TITLE ( "vodcast" ) OR ABS ( "vodcast" ) OR TITLE ( "google plus" ) OR ABS ( "google plus" ) OR TITLE ( "google+" ) OR ABS ( "google+" ) OR TITLE ( "instagram" ) OR ABS ( "instagram" ) OR TITLE ( "linkedin" ) OR ABS ( "linkedin" ) OR TITLE ( "pinterest" ) OR ABS ( "pinterest" ) OR TITLE ( "reddit" ) OR ABS ( "reddit" ) OR TITLE ( "second life" ) OR ABS ( "second life" ) OR TITLE ( "tumblr" ) OR ABS ( "tumblr" ) OR TITLE ( "weibo" ) OR ABS ( "weibo" ) OR TITLE ( "wechat" ) OR ABS ( "wechat" ) OR TITLE ( "grindr" ) OR ABS ( "grindr" ) OR TITLE ( "jack'd" ) OR ABS ( "jack'd" ) OR TITLE ( "scruff" ) OR ABS ( "scruff" ) OR TITLE ( "growlr" ) OR ABS ( "growlr" ) OR TITLE ( "okcupid" ) OR ABS ( "okcupid" ) OR TITLE ( "qq" ) OR ABS ( "qq" ) OR TITLE ( "qzone" ) OR ABS ( "qzone" ) OR TITLE ( "skype" ) OR ABS ( "skype" ) OR TITLE ( "snapchat" ) OR ABS ( "snapchat" ) OR TITLE ( "v kontakte" ) OR ABS ( "v kontakte" ) OR TITLE ( "whatsapp" ) OR ABS ( "whatsapp" ) OR TITLE ( "baidu tieba" ) OR ABS ( "baidu tieba" ) OR TITLE ( "viber" ) OR ABS ( "viber" ) OR TITLE ( "zank" ) OR ABS ( "zank" ) OR TITLE ( "hornet" ) OR ABS ( "hornet" ) OR TITLE ( "messenger" ) OR ABS ( "messenger" ) OR TITLE ( "blued" ) OR ABS ( "blued" ) OR TITLE ( "tinder" ) OR ABS ( "tinder" ) OR TITLE ( "tantan" ) OR ABS ( "tantan" ) OR TITLE ( "momo" ) OR ABS ( "momo" ) OR TITLE ( "soul" ) OR ABS ( "soul" ) OR TITLE ( "gpark" ) OR ABS ( "gpark" ) OR TITLE ( "aloha" ) OR ABS ( "aloha" ) ) AND ( TITLE ( "promot*" ) OR ABS ( "promot*" ) OR TITLE ( "prevent*" ) OR ABS ( "prevent*" ) OR TITLE ( "interven*" ) OR ABS ( "interven*" ) OR TITLE ( "implement*" ) OR ABS ( "implement*" ) OR TITLE ( "evaluat*" ) OR ABS ( "evaluat*" ) OR TITLE ( "effect*" ) OR ABS ( "effect*" ) ) AND LANGUAGE ( english ) AND PUBYEAR > 1979

1.4 WOS

#1 TI=("HIV") OR AB=("HIV") OR TI=("human immunodeficiency virus") OR AB=("human immunodeficiency virus") OR TI=("human immunodeficiency viruses") OR AB=("human immunodeficiency viruses") OR TI=("AIDS") OR AB=("AIDS") OR TI=("acquired immunodeficiency syndrome") OR AB=("acquired immunodeficiency syndrome") OR TI=("acquired immune deficiency syndrome") OR AB=("acquired immune deficiency syndrome") OR TI=("acquired immuno-deficiency syndrome") OR AB=("acquired immuno-deficiency syndrome")

#2 TI=("social media") OR AB=("social media") OR TI=("social medium") OR AB=("social medium") OR TI=("communications media") OR AB=("communications media") OR TI=("computer communication network") OR AB=("computer communication network") OR TI=("internet") OR AB=("internet") OR TI=("digital communication technology") OR AB=("digital communication technology") OR TI=("digital technology") OR AB=("digital technology") OR TI=("online") OR AB=("online") OR TI=("blogging") OR AB=("blogging") OR TI=("webcast") OR AB=("webcast") OR TI=("web") OR AB=("web") OR TI=("health information") OR AB=("health information") OR TI=("medical information") OR AB=("medical information") OR TI=("ehealth") OR AB=("ehealth") OR TI=("e-health") OR AB=("e-health") OR TI=("electronic health") OR AB=("electronic health") OR TI=("mHealth") OR AB=("mHealth") OR TI=("mobile health") OR AB=("mobile health") OR TI=("telemedicine") OR AB=("telemedicine") OR TI=("telecommunication") OR AB=("telecommunication") OR TI=("e-mail") OR AB=("e-mail") OR TI=("email") OR AB=("email") OR TI=("application") OR AB=("application") OR TI=("app") OR AB=("app") OR TI=("social web") OR AB=("social web") OR TI=("social network") OR AB=("social network") OR TI=("social networking") OR AB=("social networking") OR TI=("geosocial networking") OR AB=("geosocial networking") OR TI=("geosocial network") OR AB=("geosocial network") OR TI=("GSN application") OR AB=("GSN application") OR TI=("GSN app") OR AB=("GSN app") OR TI=("smartphone") OR AB=("smartphone") OR TI=("mobile app") OR AB=("mobile app") OR TI=("mobile application") OR AB=("mobile application") OR TI=("software application") OR AB=("software application") OR TI=("software app") OR AB=("software app") OR TI=("social software") OR AB=("social software") OR TI=("social gaming") OR AB=("social gaming") OR TI=("blog") OR AB=("blog") OR TI=("podcast") OR AB=("podcast") OR TI=("Facebook") OR AB=("Facebook") OR TI=("web log") OR AB=("web log") OR TI=("YouTube") OR AB=("YouTube") OR TI=("twitter") OR AB=("twitter") OR TI=("tweet") OR AB=("tweet") OR TI=("gay apps") OR AB=("gay apps") OR TI=("microblogging") OR AB=("microblogging") OR TI=("vodcast") OR AB=("vodcast") OR TI=("Google Plus") OR AB=("Google Plus") OR TI=("Google+") OR AB=("Google+") OR TI=("Instagram") OR AB=("Instagram") OR TI=("LinkedIn") OR AB=("LinkedIn") OR TI=("Pinterest") OR AB=("Pinterest") OR TI=("Reddit") OR AB=("Reddit") OR TI=("Second Life") OR AB=("Second Life") OR TI=("Tumblr") OR AB=("Tumblr") OR TI=("Weibo") OR AB=("Weibo") OR TI=("WeChat") OR AB=("WeChat") OR TI=("Grindr") OR AB=("Grindr") OR TI=("Jack’d") OR AB=("Jack’d") OR TI=("Scruff") OR AB=("Scruff") OR TI=("Growlr") OR AB=("Growlr") OR TI=("OkCupid") OR AB=("OkCupid") OR TI=("QQ") OR AB=("QQ") OR TI=("Qzone") OR AB=("Qzone") OR TI=("Skype") OR AB=("Skype") OR TI=("Snapchat") OR AB=("Snapchat") OR TI=("V Kontakte") OR AB=("V Kontakte") OR TI=("Whatsapp") OR AB=("Whatsapp") OR TI=("Baidu tieba") OR AB=("Baidu tieba") OR TI=("Viber") OR AB=("Viber") OR TI=("Zank") OR AB=("Zank") OR TI=("Hornet") OR AB=("Hornet") OR TI=("Messenger") OR AB=("Messenger") OR TI=("Blued") OR AB=("Blued") OR TI=("Tinder") OR AB=("Tinder") OR TI=("TanTan") OR AB=( "TanTan") OR TI=("MOMO") OR AB=("MOMO") OR TI=("Soul") OR AB=("Soul") OR TI=("Gpark") OR AB=("Gpark") OR TI=( "Aloha") OR AB=(" Aloha")

#3 TI=(promot*) OR AB=(promot*) OR TI=(prevent*) OR AB=(prevent*) OR TI=(interven*) OR AB=(interven*) OR TI=(implement*) OR AB=(implement*) OR TI=(evaluat*) OR AB=(evaluat*) OR TI=(effect*) OR AB=(effect*)

#4 (DOP=(1980-01-01/2022-12-31)) AND LA=(English)

#5 #1 AND #2 AND #3 AND #4

2. Chinese database

2.1 CNKI (中国知网)

#1 TI="艾滋病" OR TI="HIV" OR TI="人类免疫缺陷病毒" OR TI="AIDS" OR TI="获得性免疫缺陷综合征" OR AB= "艾滋病" OR AB="HIV" OR AB="人类免疫缺陷病毒" OR AB="AIDS" OR AB="获得性免疫缺陷综合征" 1980/01/01-2022/12/31

Search from the results

#2 TI="网络" OR TI="互联网" OR TI="因特网" OR TI="线上" OR TI="在线" OR TI="平台" OR TI="新媒体" OR TI="数字健康" OR TI="直播" OR TI="虚拟平台" OR TI="虚拟社区" OR TI="远程" OR TI="电子" OR TI="电脑" OR TI="手机" OR TI="邮件" OR TI="应用程序" OR TI="App" OR TI="社交软件" OR TI="手机软件" OR TI="社交媒体" OR TI="网络交友" OR TI="社交网络" OR TI="网络聊天室" OR TI="地理社交应用" OR TI="地理社交媒体" OR TI="地理社交软件" OR TI="GSN App" OR TI="交友软件" OR TI="微博" OR TI="百度" OR TI="贴吧" OR TI="Grindr" OR TI="Blued" OR TI="微信" OR TI="QQ" OR TI="Zank" OR TI="Jack’d" OR TI="Hornet" OR TI="探探" OR TI="陌陌" OR TI="Soul" OR TI="Gpark" OR TI="Aloha" OR AB="网络" OR AB="互联网" OR AB="因特网" OR AB="线上" OR AB="在线" OR AB="平台" OR AB="新媒体" OR AB="数字健康" OR AB="直播" OR AB="虚拟平台" OR AB="虚拟社区" OR AB="远程" OR AB="电子" OR AB="电脑" OR AB="手机" OR AB="邮件" OR AB="应用程序" OR AB="App" OR AB="社交软件" OR AB="手机软件" OR AB="社交媒体" OR AB="网络交友" OR AB="社交网络" OR AB="网络聊天室" OR AB="地理社交应用" OR AB="地理社交媒体" OR AB="地理社交软件" OR AB="GSN App" OR AB="交友软件" OR AB="微博" OR AB="百度" OR AB="贴吧" OR AB="Grindr" OR AB="Blued" OR AB="微信" OR AB="QQ" OR AB="Zank" OR AB="Jack’d" OR AB="Hornet" OR AB="探探" OR AB="陌陌" OR AB="Soul" OR AB="Gpark" OR AB="Aloha" 1980/01/01-2022/12/31

Search from the results

#3 TI="干预" OR AB="干预" OR TI="防治" OR AB="防治" OR TI="促进" OR AB="促进" OR TI="预防" OR AB="预防" OR TI="评价" OR AB="评价" OR TI="评估" OR AB="评估" OR TI="效果" OR AB="效果"

2.2 Wanfang(万方)

Three literature types were included in the literature search of Wanfang database: journal papers, dissertations, and conference papers.

#1 Choose “题名” for searching

(题名:(“艾滋病” or “HIV” or “人类免疫缺陷病毒” or “AIDS” or “获得性免疫缺陷综合征”) and 题名:(“网络” or “互联网” or “因特网” or “线上” or “在线” or “平台” or “新媒体” or “数字健康” or “直播” or “虚拟平台” or “虚拟社区” or “远程” or “电子” or “电脑” or “手机” or “邮件” or “应用程序” or “App” or “社交软件” or “手机软件” or “社交媒体” or “网络交友” or “社交网络” or “网络聊天室” or “地理社交应用” or “地理社交媒体” or “地理社交软件” or “GSN App” or “交友软件” or “微博” or “百度” or “贴吧” or “Grindr” or “Blued” or “微信” or “QQ” or “Zank” or “Jack’d” or “Hornet” or “探探” or “陌陌” or “Soul” or “Gpark” or “Aloha”) and 题名:(“干预” or “防治” or “促进” or “预防” or “评价” or “评估” or “效果”)) and Date:1980-*

#2 Choose“摘要”for searching

检索表达式： (摘要:(“艾滋病” or “HIV” or “人类免疫缺陷病毒” or “AIDS” or “获得性免疫缺陷综合征”) and 摘要:(“网络” or “互联网” or “因特网” or “线上” or “在线” or “平台” or “新媒体” or “数字健康” or “直播” or “虚拟平台” or “虚拟社区” or “远程” or “电子” or “电脑” or “手机” or “邮件” or “应用程序” or “App” or “社交软件” or “手机软件” or “社交媒体” or “网络交友” or “社交网络” or “网络聊天室” or “地理社交应用” or “地理社交媒体” or “地理社交软件” or “GSN App” or “交友软件” or “微博” or “百度” or “贴吧” or “Grindr” or “Blued” or “微信” or “QQ” or “Zank” or “Jack’d” or “Hornet” or “探探” or “陌陌” or “Soul” or “Gpark” or “Aloha”) and 摘要:(“干预” or “防治” or “促进” or “预防” or “评价” or “评估” or “效果”)) and Date:1980-*

#3 Combine the search results of #1 and #2

2.3 VIP (维普)

This database contains articles from 1989 onwards, so the search time interval is from 1989 to 2022.

#1 Choose “题名” from advanced search

((((((题名=“艾滋病” OR 题名=“HIV”) OR 题名=“人类免疫缺陷病毒”) OR 题名=“AIDS”) OR 题名=“获得性免疫缺陷综合征”) AND (((((((((((((((((((((((((((((((((((((((((((题名=“网络” OR 题名=“互联网”) OR 题名=“因特网”) OR 题名=“线上”) OR 题名=“在线”) OR 题名=“平台”) OR 题名=“新媒体”) OR 题名=“数字健康”) OR 题名=“直播”) OR 题名=“虚拟平台”) OR 题名=“虚拟社区”) OR 题名=“远程”) OR 题名=“电子”) OR 题名=“电脑”) OR 题名=“手机”) OR 题名=“邮件”) OR 题名=“应用程序”) OR 题名=“App”) OR 题名=“社交软件”) OR 题名=“手机软件”) OR 题名=“社交媒体”) OR 题名=“网络交友”) OR 题名=“社交网络”) OR 题名=“网络聊天室”) OR 题名=“地理社交应用”) OR 题名=“地理社交媒体”) OR 题名=“地理社交软件”) OR 题名=“GSN App”) OR 题名=“交友软件”) OR 题名=“微博”) OR 题名=“百度”) OR 题名=“贴吧”) OR 题名=“Grindr”) OR 题名=“Blued”) OR 题名=“微信”) OR 题名=“QQ”) OR 题名=“Zank”) OR 题名=“Jack’d”) OR 题名=“Hornet”) OR 题名=“探探”) OR 题名=“陌陌”) OR 题名=“Soul”) OR 题名=“Gpark”) OR 题名=“Aloha”)) AND ((((((题名=“干预” OR 题名=“防治”) OR 题名=“促进”) OR 题名=“预防”) OR 题名=“评价”) OR 题名=“评估”) OR 题名=“效果”)) AND (years:[1989 TO 2022])

#2 Choose “摘要” from advanced search

((((((摘要=“艾滋病” OR 摘要=“HIV”) OR 摘要=“人类免疫缺陷病毒”) OR 摘要=“AIDS”) OR 摘要=“获得性免疫缺陷综合征”) AND (((((((((((((((((((((((((((((((((((((((((((摘要=“网络” OR 摘要=“互联网”) OR 摘要=“因特网”) OR 摘要=“线上”) OR 摘要=“在线”) OR 摘要=“平台”) OR 摘要=“新媒体”) OR 摘要=“数字健康”) OR 摘要=“直播”) OR 摘要=“虚拟平台”) OR 摘要=“虚拟社区”) OR 摘要=“远程”) OR 摘要=“电子”) OR 摘要=“电脑”) OR 摘要=“手机”) OR 摘要=“邮件”) OR 摘要=“应用程序”) OR 摘要=“App”) OR 摘要=“社交软件”) OR 摘要=“手机软件”) OR 摘要=“社交媒体”) OR 摘要=“网络交友”) OR 摘要=“社交网络”) OR 摘要=“网络聊天室”) OR 摘要=“地理社交应用”) OR 摘要=“地理社交媒体”) OR 摘要=“地理社交软件”) OR 摘要=“GSN App”) OR 摘要=“交友软件”) OR 摘要=“微博”) OR 摘要=“百度”) OR 摘要=“贴吧”) OR 摘要=“Grindr”) OR 摘要=“Blued”) OR 摘要=“微信”) OR 摘要=“QQ”) OR 摘要=“Zank”) OR 摘要=“Jack’d”) OR 摘要=“Hornet”) OR 摘要=“探探”) OR 摘要=“陌陌”) OR 摘要=“Soul”) OR 摘要=“Gpark”) OR 摘要=“Aloha”)) AND ((((((摘要=“干预” OR 摘要=“防治”) OR 摘要=“促进”) OR 摘要=“预防”) OR 摘要=“评价”) OR 摘要=“评估”) OR 摘要=“效果”)) AND (years:[1989 TO 2022])

#3 Combine the search results of #1 and #2
